# Mast Cell and Eosinophil Activation Are Associated With COVID-19 and TLR-Mediated Viral Inflammation: Implications for an Anti-Siglec-8 Antibody

**DOI:** 10.3389/fimmu.2021.650331

**Published:** 2021-03-10

**Authors:** Simon Gebremeskel, Julia Schanin, Krysta M. Coyle, Melina Butuci, Thuy Luu, Emily C. Brock, Alan Xu, Alan Wong, John Leung, Wouter Korver, Ryan D. Morin, Robert P. Schleimer, Bruce S. Bochner, Bradford A. Youngblood

**Affiliations:** ^1^Allakos Inc., Redwood City, CA, United States; ^2^Department of Molecular Biology and Biochemistry, Research Centre, Simon Fraser University, Vancouver, BC, Canada; ^3^Division of Allergy and Immunology, Department of Medicine, Northwestern University Feinberg School of Medicine, Chicago, IL, United States

**Keywords:** COVID-19, SARS-CoV-2, Toll-like receptor, mast cell, eosinophil, Siglec-8, lirentelimab, viral inflammation

## Abstract

Coronavirus disease 2019 (COVID-19) caused by SARS-CoV-2 infection represents a global health crisis. Immune cell activation via pattern recognition receptors has been implicated as a driver of the hyperinflammatory response seen in COVID-19. However, our understanding of the specific immune responses to SARS-CoV-2 remains limited. Mast cells (MCs) and eosinophils are innate immune cells that play pathogenic roles in many inflammatory responses. Here we report MC-derived proteases and eosinophil-associated mediators are elevated in COVID-19 patient sera and lung tissues. Stimulation of viral-sensing toll-like receptors *in vitro* and administration of synthetic viral RNA *in vivo* induced features of hyperinflammation, including cytokine elevation, immune cell airway infiltration, and MC-protease production—effects suppressed by an anti-Siglec-8 monoclonal antibody which selectively inhibits MCs and depletes eosinophils. Similarly, anti-Siglec-8 treatment reduced disease severity and airway inflammation in a respiratory viral infection model. These results suggest that MC and eosinophil activation are associated with COVID-19 inflammation and anti-Siglec-8 antibodies are a potential therapeutic approach for attenuating excessive inflammation during viral infections.

## Introduction

The rapid spread of severe respiratory syndrome coronavirus 2 (SARS-CoV-2) and resulting coronavirus disease 2019 (COVID-19) pose an unprecedented global health crisis. While the majority of cases resolve with mild symptoms or no symptoms at all, some patients develop fatal complications, such as acute respiratory distress syndrome (ARDS), for which effective therapeutic strategies are urgently needed (1, 2). In these severe cases, a hyperinflammatory response or cytokine storm has been observed and is suspected to be a potential driver of pathology (3, 4). Indeed, transcriptomic profiling and histologic examination of the lungs or bronchoalveolar lavage (BAL) fluid of COVID-19 patients have revealed extensive immune cell infiltration and significantly elevated levels of cytokines, chemokines, and other proinflammatory mediators that correlate with disease severity (5–7). Evidence currently points to immune dysfunction as a potential driver of these hallmark characteristics of COVID-19. However, our understanding of the specific immune responses to SARS-CoV-2 remains extremely limited.

Innate immune sensing serves as the first line of antiviral defense and is initiated by the recognition of conserved pathogen-associated molecular patterns by pattern recognition receptors (PRRs). Single-stranded RNA (ssRNA) viruses, such as SARS-CoV-2, replicate via formation of double-stranded RNA (dsRNA) intermediates, which can be detected by Toll-like receptor (TLR) 3 and cytosolic PRRs MDA-5 and RIG-1, while ssRNA can be detected by TLR7 and TLR8 (8). Indeed, activation of immune cells via PRRs has been postulated to drive the release of proinflammatory cytokines seen in severe COVID-19 patients (8–10).

Mast cells (MCs) are tissue resident immune cells that constitute a major sensory arm of the innate immune system. They are crucially located at sites that interface with the external environment, such as the lungs and gastrointestinal tract, allowing them to be among the first cells to respond during pathogen invasion (11). MCs are equipped with TLRs and receptors for inflammatory mediators, allowing them to act as sentinels for tissue damage and pathogen exposure (12). Upon activation, MCs release preformed granules containing inflammatory mediators, vasoactive autocoids, and catalytically active MC-specific proteases, including β-tryptase, chymase, and carboxypeptidase (CPA)-3 (13). In humans, MCs are classified according to their protease content and tissue distribution, with the MC_T_ subclass expressing only tryptase and being primarily found in mucosal tissues and the MC_TC_ subclass expressing tryptase, chymase, and CPA-3 and located mainly in the skin (13). MC activation also leads to *de novo* production of cytokines and lipid mediators, including TNF, IL-6, CCL2, CCL3, prostaglandin D2 and E2, and leukotriene B4 and C4 (14, 15), many of which are now known to be associated with the cytokine storm observed in COVID-19 (5–7, 16).

MC responses to viral pathogens have not been extensively studied. Viruses can activate MCs directly or indirectly through viral or inflammatory products such as, ssRNA or dsRNA replication intermediates, complement, and cytokines (17). Many viruses have been shown to induce MC degranulation, protease release, and cytokine production, including dengue (DENV), respiratory syncytial virus (RSV), herpes simplex virus (HSV), Japanese encephalitis (JEV), Zika, and influenza (18, 19). The interactions between MCs and viruses or pathogen-derived products are complex and can result in either beneficial or detrimental outcomes (17, 18). For example, MCs have been shown to play a protective role against HSV and vaccinia virus infection (17, 19). In contrast, tryptase and chymase are elevated in plasma from patients with severe DENV infection and these MC proteases were shown to induce significant vascular leakage in peripheral tissues in response to the infection (20).

Sialic acid-binding immunoglobulin-like lectin (Siglec)-8 is an inhibitory receptor, selectively expressed on MCs and eosinophils, that inhibits MC activation and induces eosinophil death and depletion when engaged with a monoclonal antibody (mAb) (21–23). Anti-Siglec-8 mAbs have been shown to suppress immune cell infiltration, local and systemic inflammation, protease production, fibrosis, and anaphylaxis (24, 25). Clinical evaluation of lirentelimab (AK002), a humanized anti-Siglec-8 mAb, is currently underway in multiple mast cell and eosinophil-driven diseases (26, 27).

Given the pathogenic role of MCs in many inflammatory diseases and their putative role in COVID-19 pathogenesis (28–30), we sought to evaluate MC activation in SARS-CoV-2 patients and the activity of a Siglec-8 mAb in models of viral inflammation using Siglec-8 transgenic mice. Here we show that MC-derived proteases are significantly elevated in COVID-19 patient sera and lung autopsies. Surprisingly, we also found evidence of eosinophil activation in these COVID-19 patients. Stimulation of viral-sensing toll-like receptors *in vitro* and administration of synthetic viral RNA *in vivo* induced local and systemic inflammation, including cytokine elevation, immune cell airway infiltration, MC-protease production, and eosinophil-granule release. Treatment of Siglec-8 transgenic mice with an anti-Siglec-8 mAb significantly suppressed airway inflammation induced by either administration of synthetic viral RNA or infection with RSV. These data provide evidence that MC activation is a component of COVID-19 inflammation and demonstrate that targeting Siglec-8 with a mAb suppresses TLR- and RSV-mediated inflammation, supporting anti-Siglec-8 antibodies as a potential therapeutic approach for attenuating excessive inflammation during viral infections.

## Materials and Methods

### Human Serum Samples

Sera from uninfected controls (*n* = 20) and SARS-CoV-2 patients (*n* = 19) were obtained from Discovery Life Sciences Biobank (San Luis Obispo, CA, USA). The SARS-CoV-2 status of these donors was determined using the Abbott RT-PCR nasopharyngeal swab test. Serum cytokine levels were quantified using multiplex analysis Meso Scale Discovery (MSD). Mast cell activation was quantified in serum using MC-derived proteases: β-tryptase (ELH-TPSB2-1, Ray Biotech), CPA3 (CPA3, LS-F7363, LS Bio) or chymase (50-149-8059, Biomatik). Mast cell tryptase activity (mature tryptase) was determined by the Tosyl-Gly-Pro-Lys-pNA-based method (IMM001, Sigma-Aldrich) according to the manufacture's instructions. Human eosinophil-derived neurotoxin (EDN) was measured by ELISA (LS-F12507, LS Bio).

### Mice and Models of Viral Inflammation

Siglec-8 tg mice were generated as previously described (25) Siglec-8 tg mice were injected with an anti-Siglec-8 mIgG1 mAB (2E2 clone, Allakos, Inc) or isotype-matched control mIgG1 mAb (Biolegend) intraperitoneally at 5 mg/kg 3 h before poly (I:C) challenge (Invivogen). On days 1 and 2, the mice were anesthetized by isoflurane inhalation and 50 ul of PBS or poly (I:C) (1 mg/ml) was administered intratracheally. On day 3, serum, bronchoalveolar lavage (BAL) fluid, lungs and peripheral blood were isolated. Cytokines mice were measured using the multiplex Meso Scale Diagnostics (MSD) assay. For the RSV infection model, Siglec-8 tg mice were were injected with an anti-Siglec-8 mIgG1 mAB (2E2 clone, Allakos, Inc.) or isotype-matched control mIgG1 mAb (Biolegend) intraperitoneally at 5 mg/kg 4 h before being anesthetized with isoflurane and infected with 1.7 × 10^6^ pfu RSVA2 in 100μl via the intranasal route. Following infection, disease severity was assessed daily by measuring body weight. On day 7 the study was terminated, and BAL fluid was collected for differential counts and viral infectivity assay. RSV A2, an A subtype RSV, was obtained from ATCC and prepared by infecting semiconfluent layers of HEp-2 cells. When the infected monolayers exhibited approximately 80% confluence, the media was clarified and supernatant was snap frozen on dry ice.

Additional methods are described in the Supplementary Material.

## Results

### MC-Specific Proteases Are Significantly Elevated in SARS-CoV-2 Patient Serum

**Figure 1:**
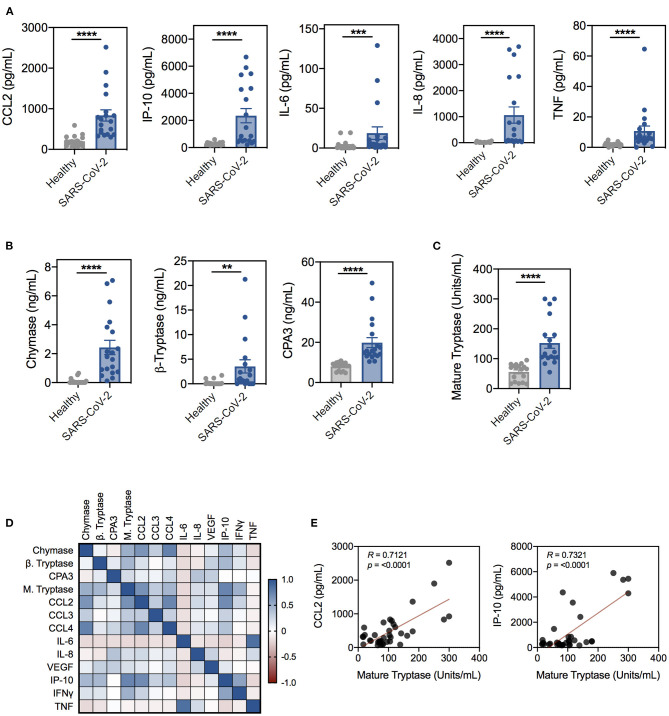
Proinflammatory cytokines and mast cell-specific proteases are significantly elevated in SARS-CoV-2-positive patient serum. **(A)** Cytokine and chemokine levels in serum from SARS-CoV-2-negative (gray; *n* = 20) or -positive (blue; *n* = 19) patients determined by the Abbott RT-PCR nasal swab test. **(B)** Levels of chymase, β-tryptase, and CPA3 in SARS-CoV-2 patient serum compared to uninfected controls. **(C)** Levels of mature tryptase in serum as determined by the Tosyl-Gly-Pro-Lys-pNA-based method. **(D)** Spearman correlation matrix and **(E)** representative Spearman correlations for MC proteases and cytokines in SARS-CoV-2 and uninfected patient serum. Data are plotted as individual donors ± SEM; **P < 0.01; ***P < 0.001; ****P < 0.0001 as determined by Mann Whitney U test.

Previous studies have demonstrated that activation of MCs and subsequent protease release contribute to virus-induced inflammation and pathology, including vascular leak, excessive airway inflammation, barrier disruption, and fibrosis (20, 31). As such, many reports have implicated MCs as putative effector cells in COVID-19 pathogenesis, however, to date, no studies have directly examined the role of MCs (32–35). To evaluate if MC activation was associated with SARS-CoV-2 inflammation, we tested the serum of SARS-CoV-2 positive patients and uninfected controls (Supplementary Table 1) for inflammatory mediators and the MC-specific proteases, chymase, β-tryptase, and CPA3. Consistent with previous reports, serum from SARS-CoV-2 patients had significantly higher levels of inflammatory mediators compared to uninfected controls, including CCL2, CCL3, CCL4, IP-10, IL-6, IL-8, VEGF, TNF, and IFN-γ (Figure 1A and Supplementary Figure 1A). We also found significantly elevated levels of chymase, β-tryptase, and CPA3 in SARS-CoV-2 patient serum, strongly suggesting systemic MC activation (Figure 1B). Consistent with increased MC activation, SARS-CoV-2 patient serum also had significantly increased levels of catalytically active, mature tryptase compared to serum from uninfected donors (Figure 1C). To gain additional insight into the association between inflammation and MC activation in SARS-CoV-2 patients, we correlated inflammatory cytokines and chemokines with MC-derived proteases. Protease levels positively correlated with levels of many inflammatory cytokines associated with COVID-19 disease severity, including IP-10, CCL2, and CCL4 (Figures 1D,E). Interestingly, the serum levels of MC-derived proteases showed little concordance (Figure 1D). The significant elevation in MC proteases but lack of correlation could represent activation of different MC populations. These data demonstrate that serum levels of MC-specific proteases are elevated in a small cohort of SARS-CoV-2 patients with generalized inflammation and suggest MC activation is a feature of COVID-19 pathogenesis.

### MC Protease and Eosinophil Granule Genes Are Increased in COVID-19 Patient Lungs

Following the characterization of MC-specific proteases in SARS-CoV-2 patient serum, we next explored the expression of these mediators in lung tissues obtained from post-mortem COVID-19 patients and control tissues from uninfected patients using publicly available RNA-seq datasets. Due to the small number of patients within each published study, we leveraged three different bulk RNA-seq datasets to generate a combined dataset with ten COVID-19 patient lungs and three lung tissue samples from uninfected individuals (36, 37). Transcriptional profiling of these samples revealed 1741 differentially expressed genes between infected lungs and controls (Supplementary Figure 1B).

**Figure 2:**
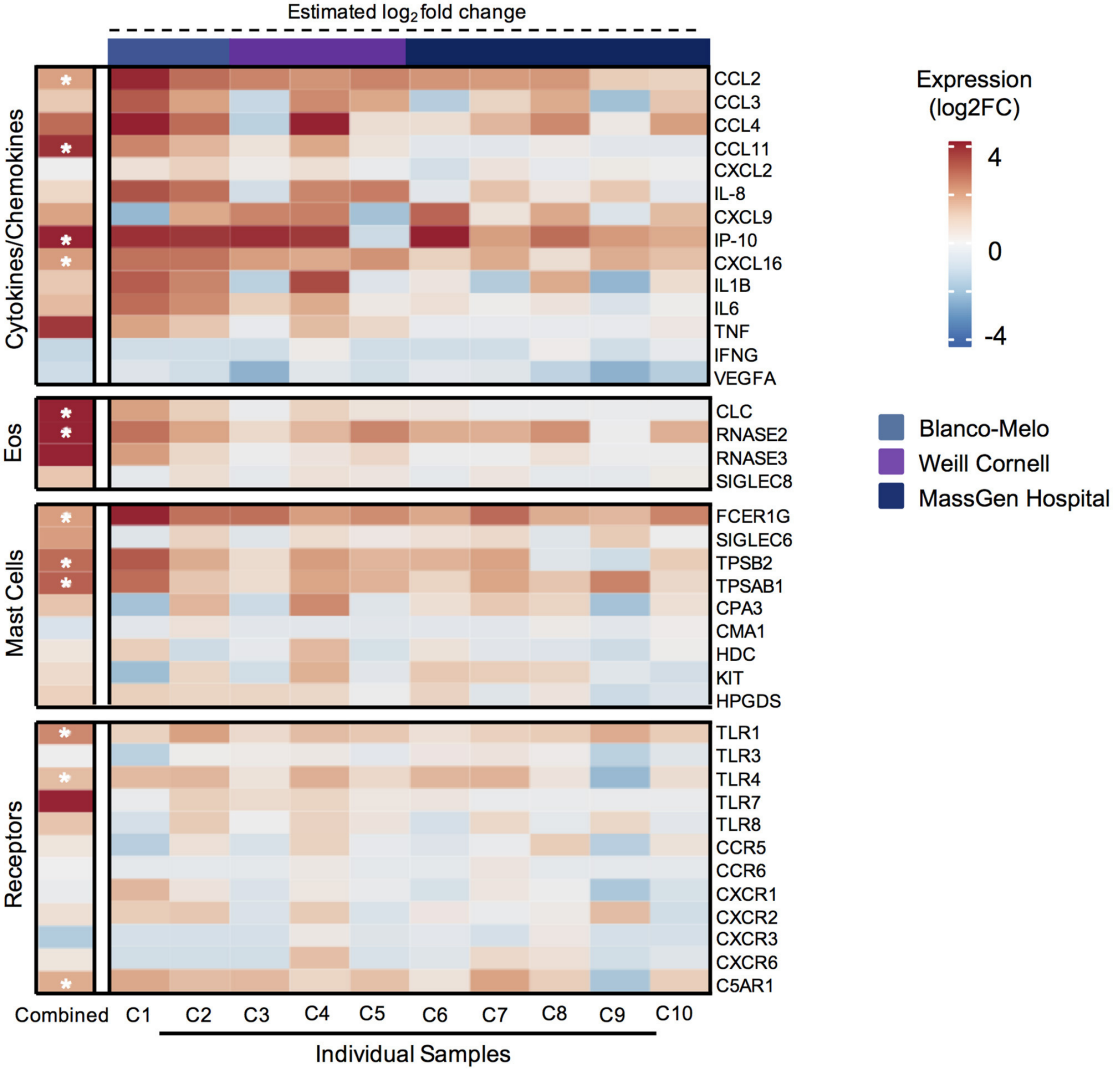
Genes associated with mast cell and eosinophil mediators are significantly elevated in lungs from COVID-19 patients. Heatmap of differentially expressed genes (DEGs), as determined by DESeq2 comparison, encoding cytokines and chemokines, eosinophil-associated genes, mast cell genes, and receptors from 3 different publicly available bulk RNA-seq data sets for COVID-19 patient lungs (*n* = 10) or uninfected control lungs (*n* = 3). (Left) Calculated differential expression for all patients is shown with asterisks (*) indicating significant DEGs (adjusted *P* < 0.1). (Right) Estimated differential expression for individual COVID-19 patient lungs was calculated using the expression of individual genes compared to the mean of all healthy samples, independent of DESeq2 modeling.

The differential expression analysis identified elevated expression of individual inflammatory cytokines and chemokines in the lungs of COVID-19 patients, including CCL2 and IP-10 (Figure 2). Consistent with the serum findings from SARS-CoV-2 patients, the MC protease genes *TPSB2* and *TPSAB1* which encode for α- and β-tryptase, respectively, were significantly elevated in lungs from COVID-19 patients, suggesting increased activation of lung MCs in COVID-19 inflammation (Figure 2). Interestingly, other MC-specific genes, such as *SIGLEC6, HDC, KIT*, and others were not increased in COVID-19 lung tissue. In addition, the MC-derived protease genes, *CPA3* and *CMA1* (chymase), were not increased in COVID-19 patient lung tissue, consistent with the tissue-specific protease expression profile found in lung MCs (Figure 2).

Given reports of eosinopenia in COVID-19 patients (38, 39), we were surprised to find that multiple eosinophil-associated granule genes were significantly upregulated in COVID-19 patient lung tissue, for example *CLC* (Galectin-10) and *RNASE2* (EDN) (Figure 2). In addition, *CCL11* (eotaxin-1), a major eosinophil chemokine, was significantly increased in COVID-19 lung patient tissue. To evaluate if these eosinophil-related proteins were also elevated in serum, we measured EDN and CCL11 in SARS-CoV-2 patient and uninfected donor serum. Consistent with the gene expression analysis, EDN and CCL11 were significantly elevated in SARS-CoV-2 patient serum (Supplementary Figure 1C). Collectively, these data show that MC-derived proteases and eosinophil-associated mediators are increased in COVID-19 lungs and/or serum and further suggest these cells could be components of COVID-19 inflammation.

### TLR Stimulation of MCs Induces Activation That Resembles Features of SARS-CoV-2 Inflammation

We next investigated potential mechanisms of MC and eosinophil activation during SARS-CoV-2 inflammation. Peripheral blood-derived human MCs and purified blood eosinophils from healthy donors had minimal expression of the cell entry receptor for SARS-CoV-2, angiotensin-converting enzyme 2 (ACE2) relative to infection-competent Calu-3 cells (Supplementary Figure 2A) (40). Consistent with the expression on peripheral blood-derived MCs, FACS-sorted healthy donor lung tissue MCs also displayed low expression of ACE2 (Supplementary Figures 2A,B). To determine if the engagement of TLRs by viral pathogens could mediate MC and eosinophil activation, we stimulated these cells with the synthetic analogs of ssRNA and dsRNA, R848 and poly (I:C), respectively. Both R848 and poly (I:C) induced significant MC activation as evidenced by increased cytokine release, including IL-8, CCL3, and CCL4 (Supplementary Figure 3A). In addition, these viral RNA analogs induced release of chymase and active mature tryptase (Supplementary Figure 3B). In contrast to MCs, only R848 induced eosinophil activation as evidenced by increased surface expression of CD69 and cytokine and chemokine production (Supplementary Figures 3C,D). However, we did not detect increased levels of EDN from TLR stimulated human blood eosinophils, suggesting eosinophil granule release is not directly induced by viral TLR stimulation (data not shown). These data demonstrate that TLR3/7/8 stimulation induces activation of MCs and to a lesser extent, eosinophils that resemble these cell-specific mediator profiles seen in SARS-CoV-2 serum and COVID-19 lung samples.

### Siglec-8 mAb Treatment *in vivo* Suppresses TLR-Driven Inflammation Induced by Poly (I:C)

**Figure 3:**
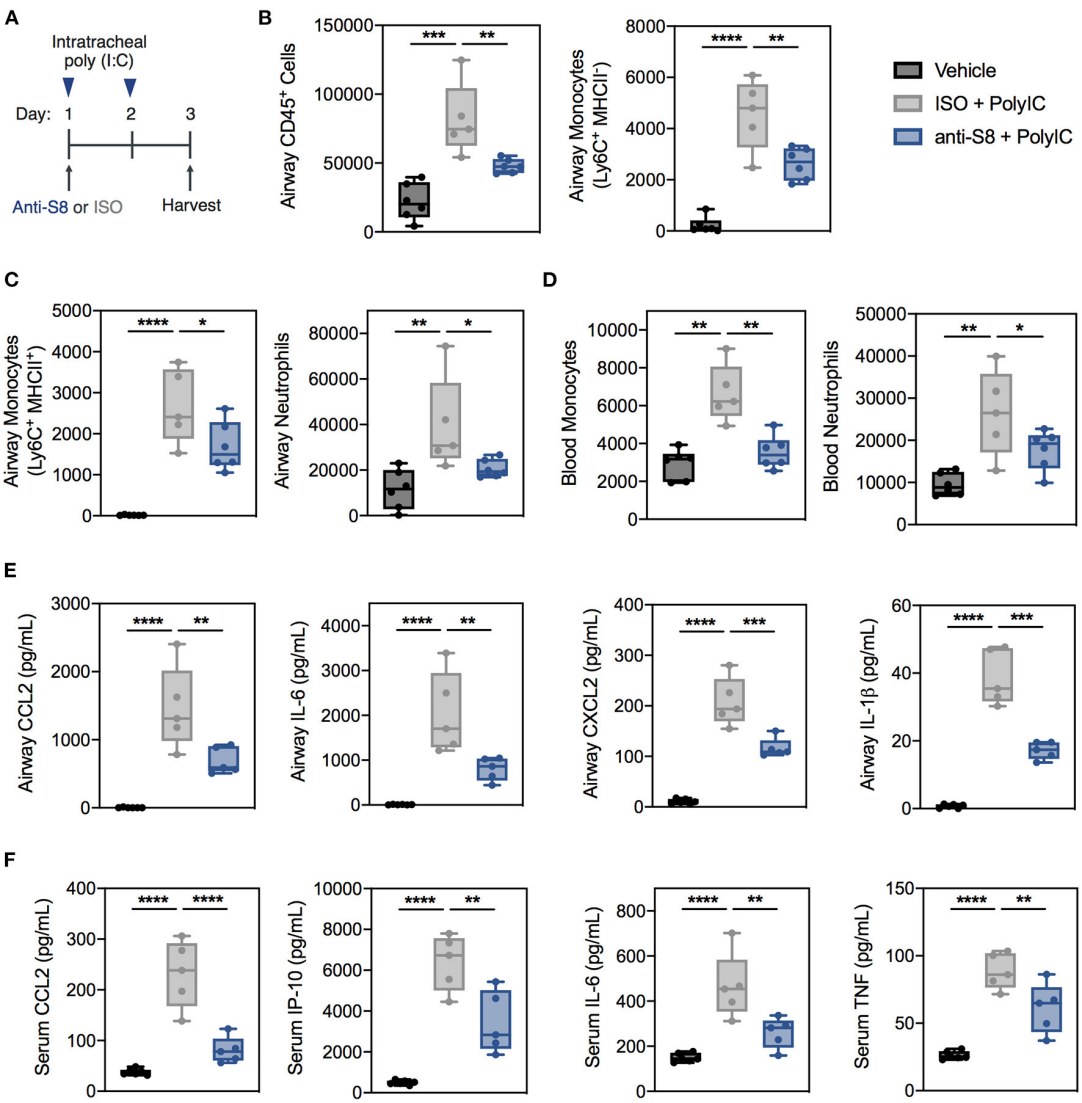
Siglec-8 mAb treatment suppresses TLR-driven inflammation induced by poly (I:C) administration. **(A)** Schematic of poly (I:C)-mediated viral inflammation model. **(B,C)** Total leukocytes, MHCII^−^ monocytes, MHCII^+^ monocytes, and neutrophils in the BAL fluid or **(D)** monocytes and neutrophils in the blood of vehicle (black), ISO + poly (I:C) (gray), or anti-S8 + poly (I:C) (blue) treated mice as determined by flow cytometry. Levels of **(E)** CCL2, IL-6, CXCL2, and IL-1b in BAL fluid or **(F)** CCL2, IP-10, IL-6, and TNF in the serum of vehicle (black), ISO + poly (I:C) (gray), or anti-S8 + poly (I:C) (blue) treated mice. Data are plotted as mean ± SEM (5–6 mice/group) and are representative of at least 2 experiments. *P < 0.05; **P < 0.01; ***P < 0.001; ****P < 0.0001 by one-way ANOVA with Tukey's multiple-comparisons test. BAL, bronchoalveolar lavage; ISO, isotype control.

Next, we wanted to examine the role of MCs and eosinophils *in vivo* in a TLR-specific viral inflammation mouse model where the anti-inflammatory activity of a Siglec-8 mAb (anti-S8) could be evaluated. To this end, we intratracheally instilled poly (I:C) to stimulate TLR3 and RIG-I-MDA5 pathways *in vivo* into Siglec-8 transgenic mice that selectively express functional Siglec-8 on mouse MCs and eosinophils (Figure 3A) (25). Administration of poly (I:C) *in vivo* has been used to mimic acute respiratory exacerbations triggered by viral infections (41, 42).

Intratracheal administration of poly (I:C) induced robust and significant airway inflammation as evidenced by infiltration of immune cells into the BAL fluid, including total leukocytes, monocytes, and neutrophils compared to vehicle control (Figures 3B,C and Supplementary Figure 4A). In addition, poly (I:C) increased blood monocytes and neutrophils (Figure 3D). One dose of anti-S8 significantly suppressed TLR-mediated infiltration of immune cells into the airway and expansion in the periphery (Figures 3B–D). Consistent with the robust immune cell infiltration into the airway, poly (I:C) administration increased cytokines and chemokines in the BAL fluid and serum (Figures 3E,F). Treatment with anti-S8 significantly reduced local and systemic cytokine and chemokine elevation induced by poly (I:C) (Figures 3E,F). These data suggest that intratracheal poly (I:C) administration results in dysregulated secretion of inflammatory cytokines and immune cell infiltration that is suppressed with administration of a Siglec-8 mAb.

### Poly (I:C)-Driven Inflammation Is Associated With MC and Eosinophil Activation That Is Suppressed With a Siglec-8 mAb

**Figure 4:**
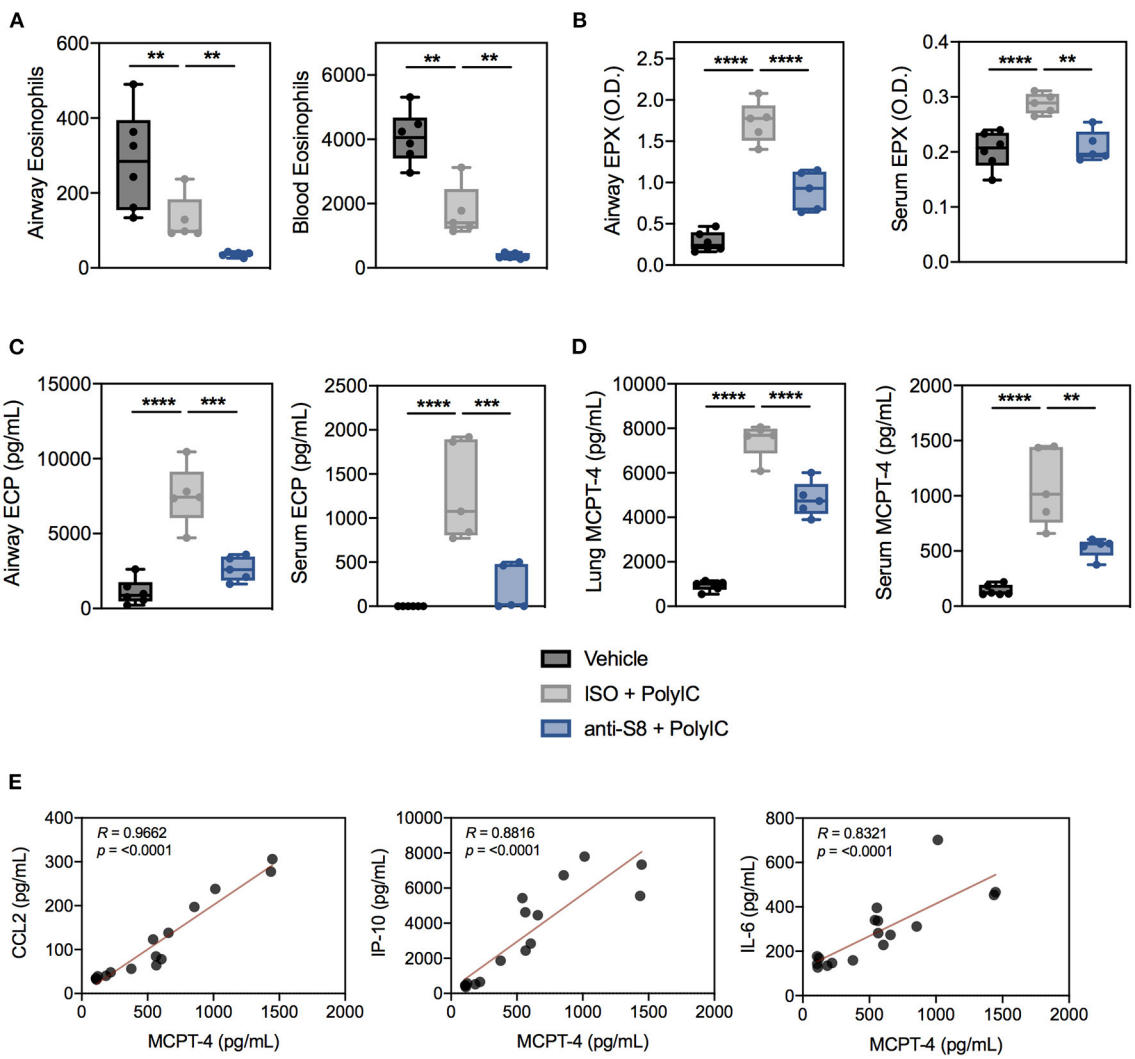
Poly (I:C)-driven inflammation is associated with aberrant MC and eosinophil activation that is suppressed with a Siglec-8 mAb. **(A)** Eosinophils in the BAL fluid and blood, **(B)** EPX levels in BAL fluid (4-fold dilution) or serum (100-fold dilution) as measured by ELISA-determined optical density (O.D.), and **(C)** ECP levels in BAL fluid or serum from vehicle (black), ISO + poly (I:C) (gray), or anti-S8 + poly (I:C) (blue) treated mice. **(D)** Levels of MCPT-4 from overnight *ex vivo* lung cultures and serum in vehicle (black), ISO + poly (I:C) (gray), or anti-S8 + poly (I:C) (blue) treated mice. **(E)** Spearman correlations for serum MCPT-4, CCL2, IP-10, and IL-6 in vehicle, ISO + poly (I:C), or anti-S8 + poly (I:C) mice. Data are plotted as mean ± SEM (5–6 mice/group) and are representative of at least 2 experiments. **P < 0.01; ***P < 0.001; ****P < 0.0001 by one-way ANOVA with Tukey's multiple-comparisons test. BAL, bronchoalveolar lavage; ISO, isotype control; MCPT-4; mast cell protease−4.

Next, we assessed if the decrease in poly (I:C)-mediated inflammation in mice treated with anti-S8 was associated with reduced MC and eosinophil activity. Interestingly, poly (I:C) administration significantly reduced eosinophil numbers in the BAL fluid and peripheral blood compared to control mice (Figure 4A). In contrast, eosinophil peroxidase (EPX) and eosinophil cationic protein (ECP) levels in the BAL fluid and serum were significantly increased in mice that received poly (I:C), suggesting TLR-driven inflammation induces activation, degranulation and subsequent reduction of eosinophils (Figures 4A–C). In support of eosinophil activation, CCL11 levels in the BAL fluid were significantly increased in mice administered poly (I:C) (Supplementary Figure 4B). Consistent with its known eosinophil depleting activity, anti-S8-treated mice had significantly reduced eosinophils in the BAL fluid and blood and decreased levels of EPX and ECP compared to isotype control mAb-treated mice administered poly (I:C) (Figures 4A–C).

To evaluate MC activation, we quantified levels of the MC-specific mediators, mast cell proteases−1 and−4 (MCPT-1 and MCPT-4) in *ex vivo* cultured lung tissue and serum in poly (I:C)-driven inflammation. Among the murine chymases, MCPT-4 is most likely the functional counterpart of human chymase because it has similar substrate specificity and proteoglycan-binding properties (43). Intratracheal poly (I:C) administration markedly increased MCPT-4, but not MCPT-1 levels in the lung and serum, indicative of MC activation (Figure 4D, data not shown). Anti-S8 treatment significantly reduced lung and serum MCPT-4 levels compared to isotype control mAb-treated mice, consistent with MC inhibition (Figure 4D). Lastly, to determine if MC protease levels were associated with proinflammatory cytokines in our poly (I:C) inflammation model, we correlated serum MCPT-4 levels with CCL2, IP-10, and IL-6. As was seen in human serum (44, 45), MC protease levels significantly correlated with many proinflammatory cytokines associated with poly (I:C) inflammation (Figure 4E). These data demonstrate that TLR-mediated inflammation driven by synthetic viral RNA induces mouse MC and eosinophil activation that is suppressed with an anti-Siglec-8 mAb.

### Siglec-8 mAb Treatment Reduces Disease Severity and Airway Inflammation Induced by RSV Infection

**Figure 5:**
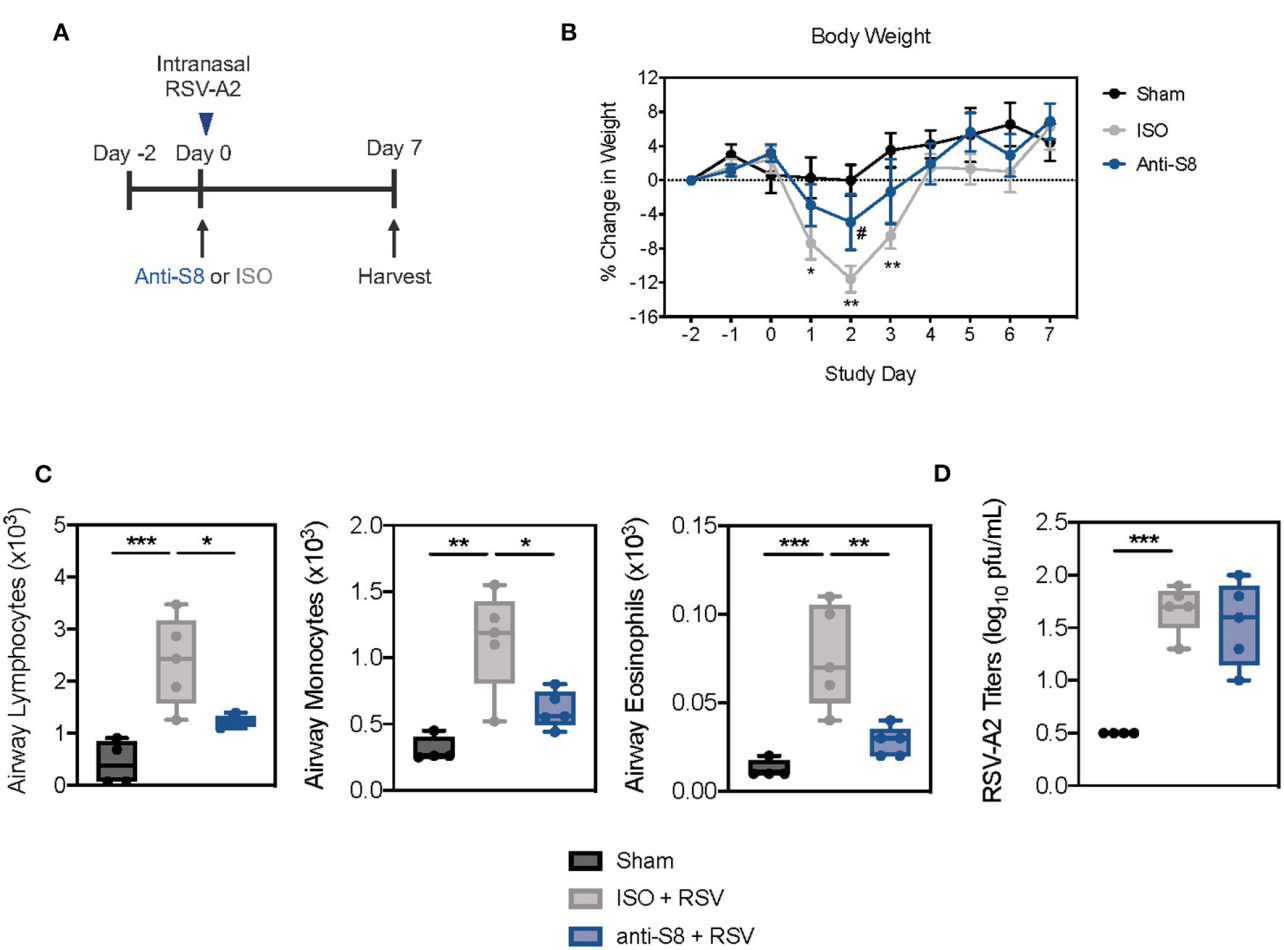
Siglec-8 mAb treatment reduces RSV-mediated airway inflammation without modulating viral titer. **(A)** Schematic of RSV-driven infection model in Siglec-8 transgenic mice. **(B)** Percent change in body weight from day−2 to day 7 in sham (black), ISO + RSV (gray), or anti-S8 + RSV (blue) treated mice. **(C)** Numbers of airway lymphocytes, monocytes, and eosinophils or **(D)** viral titer in BAL fluid on day 7 post infection of sham (black), ISO + RSV (gray), or anti-S8 + RSV (blue) treated mice. Data are plotted as mean ± SEM (4–5 mice/group). *P < 0.05; **P < 0.01; ***P < 0.001; #*P* < 0.05 (ISO vs. anti-S8, **B**) by one-way ANOVA with Tukey's multiple-comparisons test. BAL bronchoalveolar lavage, ISO isotype control.

To determine if an anti-Siglec-8 mAb could also suppress inflammation driven by human respiratory viruses, we infected Siglec-8 transgenic mice with RSV (Figure 5A). RSV is a major viral pathogen of infants and adults and has been shown to activate MCs and eosinophils through PRRs (46, 47). Mice infected with RSV demonstrated significant weight loss on days 1–3 post infection (dpi) compared to sham-treated mice (Figure 5B). Anti-S8 treatment significantly improved RSV infection-associated weight loss. Infection with RSV also induced significant immune cell airway infiltration, including increased numbers of lymphocytes, monocytes, and eosinophils in the BAL fluid (Figure 5C). Consistent with reduced weight loss seen in anti-S8-treated mice, airway inflammation was significantly decreased (Figure 5C). However, RSV viral titers were similar in isotype control- and anti-S8-treated mice (Figure 5D), suggesting that the reduction in cellular inflammation did not compromise protection against RSV infection. These data demonstrate that anti-Siglec-8 mAb-treatment can suppress RSV-induced lung inflammatory cell accumulation without worsening viral infection.

## Discussion

A hyperinflammatory response mediated by dysregulated immune cells is considered to play a detrimental role in the progression of COVID-19. While the majority of studies have implicated monocytes and macrophages as primary drivers of aberrant inflammation, very few studies have examined the role of other innate immune cells in COVID-19 inflammation (48). In the current study, we evaluated serum from SARS-CoV-2 positive patients and publicly available RNA-seq data from diseased patients to examine the presence of MCs in COVID-19 inflammation. We found significant evidence of MC activation in the serum of SARS-CoV-2 patients and COVID-19 patient lung tissue by examining the levels and expression of MC-specific proteases. The levels of these proteases also significantly correlated with inflammatory cytokines implicated in COVID-19 disease severity, including CCL2 and IP-10, suggesting MC activity could be associated with a heightened immune response. In support of this, post-mortem lung biopsies of COVID-19 patients showed increased density of perivascular and septal MCs compared to controls (30). Recent evidence also indicates that MCs can play a role in initiating hyperinflammation or cytokine storm in multiple disease settings (18, 49, 50). Our findings also suggest systemic MC activation is associated with COVID-19 inflammation since we saw signatures of elevated proteases thought to be released from different MC populations. Indeed, multiorgan clinical manifestations have been noted in some COVID-19 patients and our data supports evaluation of MC activation in these patients in the future (51).

MCs are key effector cells that regulate acute and chronic inflammatory responses through the release of preformed and *de novo* synthesized mediators in response to a diverse array of activating stimuli. Of these mediators, the MC-derived proteases, β-tryptase, chymase, and CPA-3 are considered to be specific for MCs and indicative of the activation state of MCs (52, 53). These proteases induce broad biological responses, including proliferation and contraction of smooth muscle cells, cleavage of angiotensin (ang)-I, and activation of peptides and enzyme precursors, such as matrix metalloproteinases, TGF-β, kallikrein (which produces bradykinin) and IL-18 (43). As such, amplified release of these enzymes is implicated in the pathogenesis of numerous diseases, as well as detrimental effects in viral infections (20, 54). Increased levels of chymase and tryptase are seen in severe DENV and JEV infection and contribute to the development of vasculopathy and thrombocytopenia (20, 55). Evidence of systemic vasculopathy, angiogenesis, and coagulopathy has also been found in COVID-19 patients and is suspected to contribute to pathogenesis (56). While our data do not directly compare vascular factors, elevated levels of chymase and tryptase have been directly linked to virus-induced vasculopathy and thrombotic microangiopathies (20).

Cellular entry through ACE2 is the dominant mechanism for SARS-CoV-2 (40, 57). However, studies have failed to directly link hyperinflammation with the expression profile of ACE2 on immune cells. In support of this, we detected low expression of ACE2 on MCs and eosinophils, compared to infection-permissive Calu-3 cells. Therefore, rather than direct infection, it is likely is that secondary activation of innate immune cells like MCs and eosinophils via PRRs contributes to the release of inflammatory cytokines. Indeed, innate sensors that recognize viral RNA, including TLR3/7/8, become overstimulated in response to SARS-CoV, and *in vivo* administration with viral RNA analogs induce barrier damage to the airways upon TLR activation (42, 58). We observed that TLR stimulation with synthetic viral RNA directly activated MCs *in vitro* and induced features of hyperinflammation *in vivo*, including systemic and local inflammatory cytokine production, infiltration of neutrophils and monocytes in the airway, and production of the MC-protease, MCPT-4.

Interestingly, our gene expression analysis of post-mortem COVID-19 patient lungs revealed that the eosinophil associated genes, *CLC, RNASE2* (EDN), and *CCL11* were some of the most upregulated genes in COVID-19 lung tissue. An elevation of these genes in lung tissue was striking given that eosinopenia has been a common finding in patients admitted with COVID-19 (38, 39). CCL11 has been shown to induce degranulation and EDN release from human eosinophils and contribute to cytolysis of eosinophils in mice which could potentially explain the counterintuitive finding of eosinopenia and elevated secondary granules in COVID-19 patients (59–61). In support of this, mice exposed to synthetic viral RNA *in vivo* displayed blood and airway eosinopenia, increased levels of CCL11, and elevated expression of EPX and ECP, highly suggestive of activation. Nonetheless, the precise role of eosinophils in COVID-19 inflammation remains unclear, with reports of elevated and activated peripheral eosinophils in severe cases of COVID-19 (62–64). We found that human eosinophils directly responded to TLR7/8 stimulation using R848 by producing inflammatory cytokines, consistent with previous studies (65). Neither TLR3 nor TLR7/8 stimulation induced EDN release, suggesting alternative modes of activation are required to induce secretion of secondary granules from human eosinophils. Although EDN and CLC are most abundantly expressed in eosinophils, they have also been found in other leukocytes, albeit at much lower quantities, which may account for the increased expression seen in COVID-19 patient samples (66). While the role of EDN in COVID-19 has not been widely described, it has previously been associated with neuroinflammatory disease and neuronal cell death (40). It is therefore tempting to speculate that the elevated levels of EDN observed in COVID-19 patients may contribute to neurological manifestations found in some COVID-19 patients.

There are currently a limited number of effective therapeutic strategies to treat excessive inflammation associated with viral infections, such as COVID-19. Using a well-established model of poly (I:C)-induced lung inflammation, we examined the therapeutic potential of targeting MCs and eosinophils using a Siglec-8 mAb. Anti-Siglec-8 mAbs have previously been shown to reduce immune cell infiltration, cytokine production, and tissue damage in chronic non-allergic airway models of chronic obstructive pulmonary disease and idiopathic pulmonary fibrosis (24). However, to date, the activity of an anti-Siglec-8 mAb has not been evaluated in a model of TLR-mediated inflammation. Treatment with anti-S8 significantly suppressed immune cell infiltration, local and systemic inflammation, and levels of MC-derived proteases induced by synthetic viral RNA. Given that both MCs and eosinophils have been shown to play an important role in viral clearance with certain pathogens (7, 17, 18), we also investigated the effects of an anti-Siglec-8 mAb in a murine model of RSV infection. Anti-Siglec-8 mAb treatment significantly decreased RSV-induced lung inflammation without hampering antiviral immunity, suggesting MC inhibition and eosinophil depletion via anti-Siglec-8 is an attractive approach for suppressing viral inflammation without compromising host defense. Moreover, since over 80% of asthma exacerbation episodes are associated with viral infections (67), these data highlight the potential use of anti-Siglec-8 mAbs as an approach to treat virus-induced asthma exacerbations.

While ITIM-containing receptors, such as Siglec-8, are mainly thought to inhibit immunoreceptor tyrosine activation motifs (ITAM)-bearing receptors, these data and previously published findings suggest Siglec-8 can inhibit both ITAM- and non-ITAM-bearing receptors on MCs, including FcεRI, ST2 (IL-33 receptor), MRGPRX2, and TLR (22–24, 68). Similarly, murine Siglec-E can inhibit TLR activation in a MyD88-dependent manner by decreasing NF-κB activation and the secretion of proinflammatory mediators (69). Future studies will focus on further characterizing the intracellular pathways induced by Siglec-8 ligation that lead to inhibition of MC responses.

This study has several limitations. The cohort of individuals used for serum collection and RNA-seq analyses was small and lacked robust clinical information, including disease severity, comorbidities, and patient outcomes. Follow up studies are needed on a well-characterized patient population. In addition, the blood-derived MCs and eosinophils used in these studies may not completely reflect the function and phenotype of mature diseased human tissue lung MCs and eosinophils. Lastly, the use of viral RNA mimetics or RSV to study MC and eosinophil responses to SARS-CoV-2 may not fully recapitulate the complexity of COVID-19.

The data presented here suggest that systemic MC and eosinophil activation play a role in the inflammatory mechanisms underlying disease pathogenesis of COVID-19. Importantly, these data also demonstrate that targeting Siglec-8 with an antibody reduces TLR-mediated inflammation and suggest that Siglec-8 mAbs, such as liretelimab, could be used as a means of limiting immune cell infiltration and excessive inflammation in severe viral infection.

## Data Availability Statement

The raw data supporting the conclusions of this article will be made available by the authors, without undue reservation.

## Ethics Statement

The studies involving human participants were reviewed and approved by Discovery Life Biosciences. The patients/participants provided their written informed consent to participate in this study. The animal study was reviewed and approved by Murigenics Inc., 941 Railroad Ave, Vallejo, CA 94592. Aragen Biosciences, 380 Woodview Ave, Morgan Hill, CA 95037.

## Author Contributions

SG, JS, MB, TL, EB, AX, AW, JL, and WK conducted the experiments. SG, JS, MB, and BY designed the experiments. KC and RM analyzed the RNA-seq data. BY, RS, and BB wrote the paper. All authors contributed to the article and approved the submitted version.

## Conflict of Interest

JS, SG, MB, TL, EB, AX, AW, JL,WK, and BY are employees of Allakos and/or own stock options in Allakos. BB and RS did not perform any of the experiments but are paid consultants on the scientific advisory board of Allakos, Inc., and own stock in Allakos. BB and RS are coinventors on existing Siglec-8–related patents and thus may be entitled to a share of royalties received by Johns Hopkins University from Allakos, Inc. on the potential sales of such products. BB and RS are also cofounders of Allakos, which makes him subject to certain restrictions under university policy. The remaining authors declare that the research was conducted in the absence of any commercial or financial relationships that could be construed as a potential conflict of interest.
