# Supplementary material for: Mast Cell and Eosinophil Activation Are Associated With COVID-19 and TLR-Mediated Viral Inflammation: Implications for an Anti-Siglec-8 Antibody
Source: Front Immunol. 2021 Mar 10;12:650331. doi: 10.3389/fimmu.2021.650331 (PMC7988091; doi:10.3389/fimmu.2021.650331)
Supplement: Supplementary file 1 [file Data_Sheet_1.pdf]

Supplementary Material

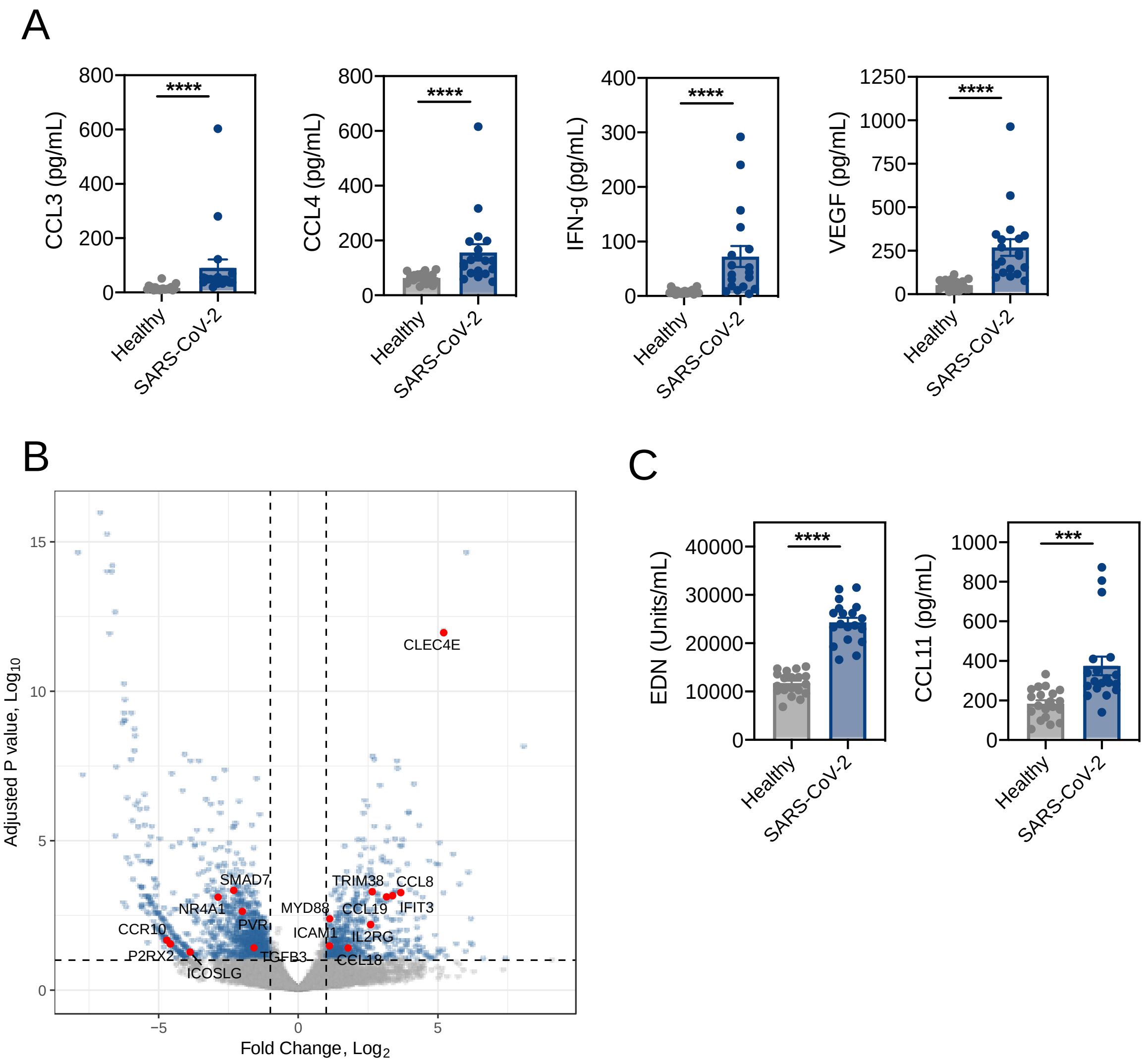

Supplementary Figure 1. Pro-inflammatory cytokines and eosinophil-associated granules are significantly elevated in SARS-CoV-2 patient serum. (A) Cytokine and chemokine levels in serum from SARS-CoV-2 negative (gray; n=20) or positive (blue; n=19) patients determined by the Abbott RT-PCR nasal swab test. (B) Volcano plot of differentially expressed genes in COVID-19 lungs (n=10) compared to uninfected control lungs (n=3) (C) Levels of eosinophil-derived neurotoxin (EDN) and CCL11 in serum from SARS-CoV-2 negative (gray; n=20) or positive (blue; n=19) patients. Differentially expressed genes were determined by (|log2 fold change| > 1 and Benjamini-Hochberg adjusted p-value < 0.1). Data are plotted as individual donors +/- SD; ** p = <0.01; *** p = <0.001; **** p = <0.0001 as determined by Mann Whitney U test.

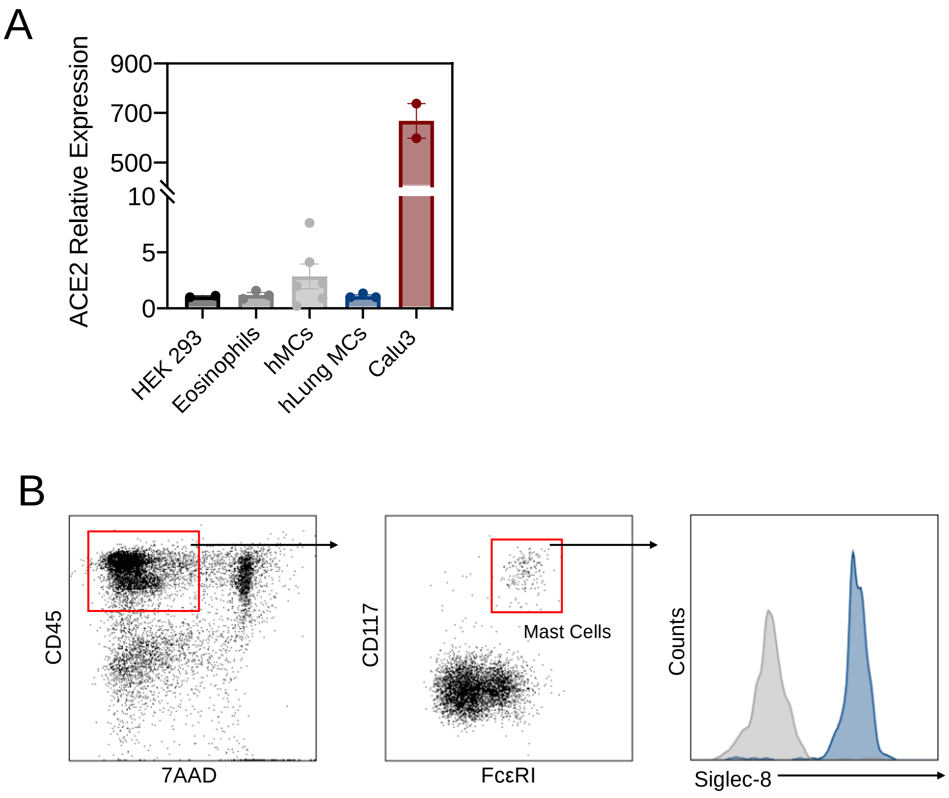

Supplementary Figure 2. Human eosinophils and mast cells have low expression of ACE2. (A) Relative gene expression of ACE2 on HEK293 cells (black), eosinophils (dark gray), blood derived human MCs (light gray), human lung tissue MCs (blue), and Calu-3 cells (red) determined by qRT-PCR. (B) Gating strategy for sorting human MCs from lung tissue. MCs were stained with either an

isotype negative control (gray) or anti-Siglec-8 mAb (blue). Data are plotted as individual donors (n = 2-6) +/- SD

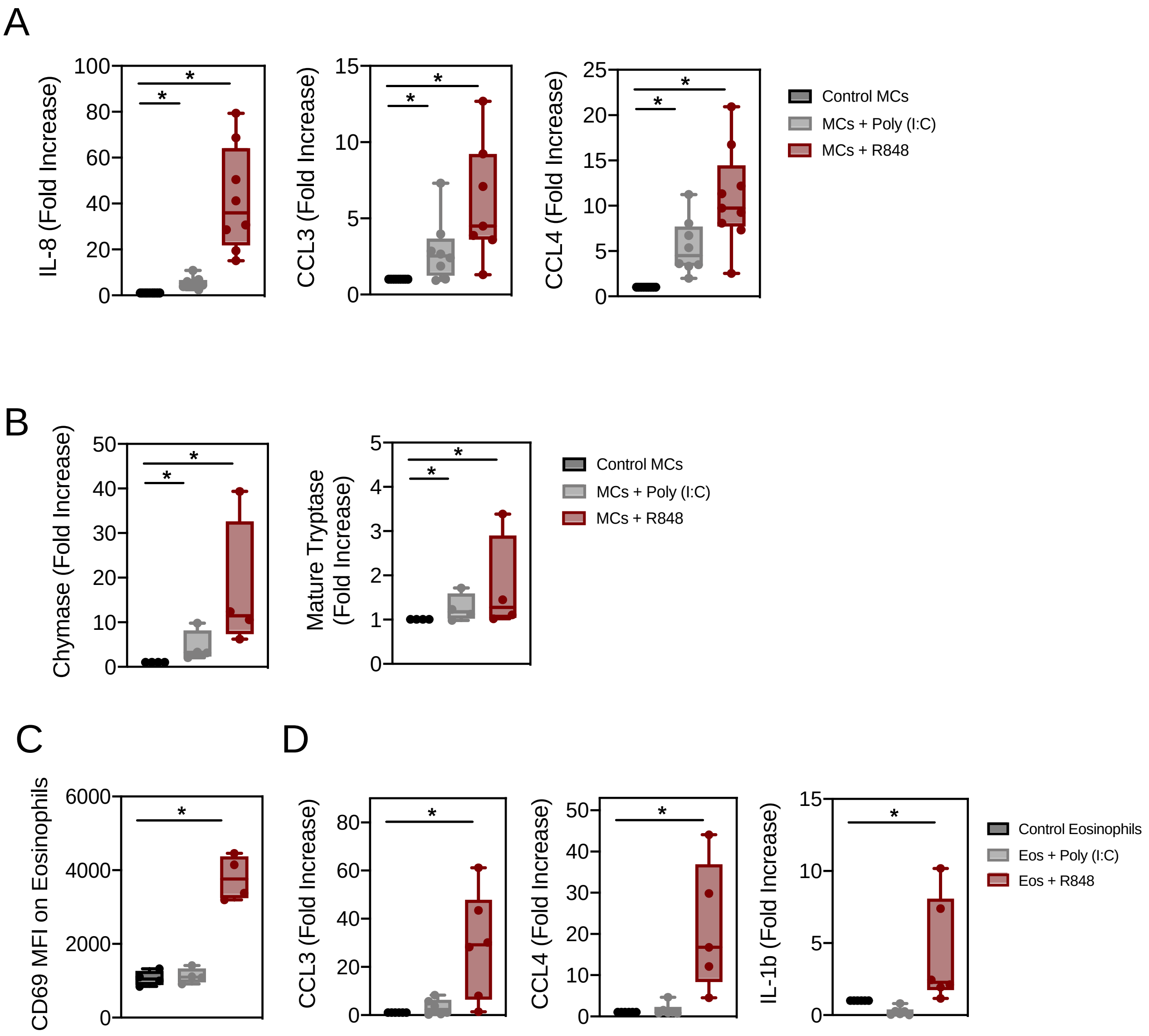

Supplementary Figure 3. TLR stimulation with poly (I:C) and R848 in vitro directly induces human mast cell and blood eosinophil activation. Fold induction of (A) cytokines and chemokines and (B) MC-derived proteases from supernatants of overnight cultured unstimulated human MCs (black) or stimulated with poly (I:C) (gray) or R848 (red). (C) Expression of CD69 on the surface of eosinophils as determined by flow cytometry and (D) fold induction of cytokines and chemokines from supernatants of overnight cultured unstimulated human blood eosinophils (black) or stimulated with poly (I:C) (gray) or R848 (red). Levels of mediators that were at least ≥ 2 pg/mL were

normalized to unstimulated and plotted as fold increase. Data are plotted as individual donors (n = 5-8) +/- SD; * p = <0.05 as determined by Mann Whitney U test.

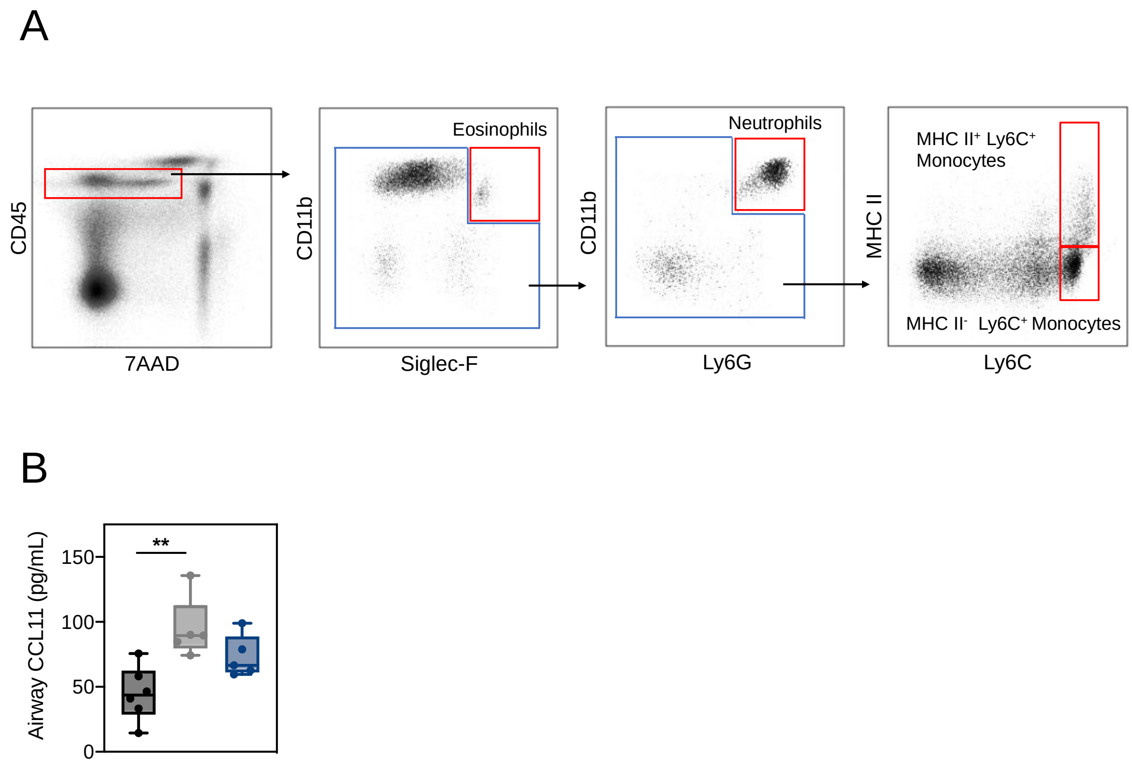

Supplementary Figure 4. Flow cytometry gating strategy for immune cells in BAL fluid. (A) Eosinophils: $7AAD^-$ $CD45^+$ $CD11b^+$ SSC^{Hi} Siglec-F^+; Neutrophils: $7AAD^-$ $CD45^+$ $Ly6C^-$ $Ly6G^+$; Monocytes: $7AAD^-$ $CD45^+$ $Ly6G^-$ $Ly6C^{Hi}$ $MHCII^{+\ or\ -}$ (B) Levels of CCL11 in the BAL fluid of vehicle (black), ISO + poly (I:C) (gray), or anti-S8 + poly (I:C) (blue) treated mice. Data are plotted as mean ± SEM (5-6 mice/group) and are representative of at least 2 experiments. ** p <0.01 by one-

way ANOVA with Tukey's multiple-comparisons test. BAL bronchoalveolar lavage, ISO isotype control.

Supplementary Table 1. COVID Patient Demographics

Patient	AGE	GENDER	RACE	COVID TEST DATE	Collection Date	PCR TEST Type
COVID1	63	Female	Unknown	3/16/20	3/16/20	Abbott
COVID2	40	Female	White	3/17/20	3/25/20	Abbott
COVID3	71	Male	White	3/29/20	3/31/20	Abbott
COVID4	24	Female	Black	4/1/20	4/2/20	Abbott
COVID5	59	Female	Black	3/31/20	4/14/20	Abbott
COVID6	56	Female	White	4/6/20	4/9/20	Abbott
COVID7	65	Female	White	4/24/20	4/26/20	Abbott
COVID8	71	Male	White	4/5/20	4/9/20	Abbott
COVID9	65	Male	White	4/26/20	4/28/20	Abbott
COVID10	68	Male	White	4/23/20	4/27/20	Abbott
COVID11	23	Female	Black	5/14/20	5/15/20	Abbott
COVID12	70	Male	White	5/11/20	5/15/20	Abbott
COVID13	29	Male	White	5/14/20	5/15/20	Abbott
COVID14	66	Female	Black	5/18/20	5/18/20	Abbott
COVID15	56	Female	Black	5/1/20	5/18/20	Abbott
COVID16	58	Male	White	5/29/20	5/29/20	Abbott
COVID17	62	Male	White	5/12/20	5/14/20	Abbott
COVID18	64	Male	White	5/14/20	5/15/20	Abbott
COVID19	29	Female	Black	5/18/20	5/18/20	Abbott
HV1	28	Female	Black	NA	3/27/20	NA
HV2	60	Male	White	NA	4/12/20	NA
HV3	26	Female	Black	NA	4/8/20	NA
HV4	86	Female	White	NA	4/15/20	NA
HV5	25	Female	White	NA	9/30/19	NA
HV6	72	Female	Black	NA	3/19/19	NA
HV7	32	Male	White	NA	10/1/19	NA
HV8	54	Male	White	NA	8/6/18	NA
HV9	34	Male	White	NA	10/1/19	NA
HV10	65	Male	White	NA	9/14/20	NA
HV11	41	Male	Asian	NA	9/14/20	NA
HV12	25	Male	Hispanic	NA	9/14/20	NA
HV13	48	Male	Asian	NA	9/14/20	NA
HV14	58	Female	White	NA	9/14/20	NA
HV15	64	Male	White	NA	9/14/20	NA
HV16	53	Male	Unknown	NA	9/14/20	NA
HV17	57	Male	White	NA	9/14/20	NA
HV18	74	Male	White	NA	9/14/20	NA
HV19	62	Male	Asian	NA	9/14/20	NA

HV20	48	Female	White	NA	9/14/20	NA

COVID, SARS-CoV-2-postive; HV, healthy volunteer

Supplementary Materials and Methods

RNA-sequencing data processing and analysis

Raw RNA-seq data was obtained from three studies through the NCBI Sequence Read Archive: Blanco-Melo (GSE147507), GSE151803, and GSE150316. Reads were aligned to GRCh38 with STAR v2.6.1a.[22] and multiple runs from the same sample were merged. PCR & optical duplicates were marked using Picard Tools v2.20.4 (Broad Institute). Gene counts were summarized using featureCounts from the subread package v1.6.3.[23] and batch effects resulting from different studies were minimized using ComBat-Seq. DESeq2 v1.22.2 was utilized to obtain differentially expressed genes (p.adj < 0.1) and a variance-stabilized matrix for visualization of individual samples. Heatmaps were generated using ComplexHeatmap v2.5.4 and data were restricted to a maximum absolute $\log_2$fold change of 4 for visualization.

Human peripheral blood derived MCs and stimulation

Healthy donor peripheral blood cells were isolated from residual cells in the leukocyte reduction chamber (TrimaAccel). CD34+ progenitor cells were isolated using the CD34 MicroBead Kit UltraPure human kit (Miltenyi Biotec) and cultured as previously described (Saito, H et al. 2006). After 7 weeks in culture, cells were maintained IMDM (Gibco) supplemented with 5% FBS (Hyclone), 55 μM β-Mercaptoethanol, 100ng/mL SCF (Peprotech), and 50ng/mL IL-6 (Peprotech). MCs were cultured in IMDM supplemented with 5% FBS and stimulated with 10 μg/ml of poly (I:C) (Invivogen) or 10 μg/ml R848 (Invivogen) overnight. Levels of cytokines and chemokines in supernatant were measured by MSD.

Human lung tissue MC isolation and RT-PCR

Fresh human lung tissue was procured and provided by the NCI Cooperative Human Tissue Network (CHTN) from subjects with no previous history of chronic lung disease, as approved by the Vanderbilt University Institutional Review Board (IRB# 031078 and 010294). The tissue was enzymatically and mechanically dissociated using the gentleMACs™ Dissociator (Miltenyi Biotec), according to manufacturer's protocol. Tissue was minced into 2 mm pieces and incubated at 37°C for one hour in digestion solution containing proprietary enzymes. Before, during, and after incubation, tissue was mechanically disrupted and run through a 70-micron filter to obtain single cells. Cells were then treated with RBC lysing buffer, washed in PBS and resuspended in RPMI 1640+10% Low IgG FBS. Immediately after digestion, cell viability was examined using flow cytometry. MCs were stained and sorted (CD45+ 7AAD- CD117+ IgER+) into RNAlater with over 95% purity. RNA was isolated from eosinophils, MCs, and Calu-3 cells according to methods provided by the manufacturer (Qiagen). cDNA was synthesized according to the manufacturer's protocol (Thermo Fisher Scientific), and qPCR was performed using SYBR green (Thermo Fisher Scientific) and predesigned

gene-specific primers for GAPDH and ACE2 (IDT). Relative gene expression was calculated using the ΔΔCt method.

Human peripheral blood eosinophil isolation and stimulation

Eosinophils were directly isolated from whole blood using the EasySep direct human eosinophil isolation kit (STEMCELL technologies Inc) with purity >97%. For stimulation experiments, cells were stimulated with 10ug/ml of poly (I:C) or R848 overnight. Supernatant was collected for cytokine analysis by MSD and cells were stained for cell surface expression of the activation marker CD69 using flow cytometry.

Flow cytometry analyses of blood and tissue

Approximately 1–5×10^6 cells were preincubated with CD16/32 antibody to block nonspecific binding. Cells were then incubated at 4°C for 10 minutes with staining antibody panels, washed, and fixed in 2% paraformaldehyde. Data acquisition was performed using a NovoCyte flow cytometer (Acea Biosciences) and FlowJo was used for data analysis. The following antibodies were purchased from eBioscience or Biolegend (clone indicated in brackets): CD3-BV650 (17A2), CD8-APC (53-6.7), CD11b-BV605 (M1/70), CD45-BV785 (30-F11), CD117-SB436 (2B8), CD206 PECy7 (MMR), F4/80-FITC (BM8), F4/80-PECy7 (BM8), Ly6G-BV510 (1A8), Ly6C-APCCy7 (HK1.4), MHCII-(IA/E)- AF488, SiglecF-PE (S17007L), FcER1-FITC (MAR-1), and Siglec-8 AF647 (1H10 clone, Allakos, Inc.). Gating strategy for cells from mouse BAL fluid and blood is shown in Supplemental Fig 3.

ECP, EPX, and MCPT-4 quantification

Eosinophil cationic protein (ECP) levels in BAL and serum were quantified using an ELISA (Cusabio, E11799m-96) and samples were diluted 5X prior to performing the assay. Eosinophil peroxidase (EPX) levels in BAL and serum were measured as previously described (Ochkur et. al 2012) (LS-F20146, LS Bio). BAL samples were diluted 4X and serum samples were diluted 100X to ensure the samples were within the threshold of detection. mMCPT4 levels were quantified in the lungs of poly (I:C) challenged or sham mice by harvesting the lungs on day 3, followed by overnight culture in RPMI and collection of supernatants. Serum was also collected on day 3. Supernatants and serum were analyzed for mMCPT4 (LSBio, LS-F55860-1) by ELISA and according to the manufacturer's instructions.

Differential cell counts and viral infectivity assay

BAL fluid was collected from lungs by perfusing the lung twice with sterile HBSS (Gibco). The BAL was centrifuged at 500xg for 5 minutes and a 100μl of supernatant was collected for the viral infectivity assay. The BAL cell pellet was resuspended in Pharmalyse Buffer (BD Biosciences) to lyse RBCs followed by cytospins stained with Geimsa to obtain differential cell counts. BAL supernatant was used to determine viral load using plaque assay as previously described (Hasan, S. et al *The Journal of Infectious Diseases*, Volume 189, Issue 10, 15 May 2004). Results of plaque assays were reported in $\log_{10}$ plaque-forming units per milliliter, with 0.5 $\log_{10}$ pfu/mL as the lowest limit of detection.

Statistical analysis

To determine statistical significance, nonparametric Mann-Whitney U test, unpaired 2-tailed t test, 2-tailed t test with Holm-Šídák's posttest, or 1-way ANOVA with Tukey's posttest for multiple comparisons was performed using Prism (GraphPad Software). A *P*-value of 0.05 or less was considered significant.
